# Supplementary material for: Stirred suspension bioreactors maintain naïve pluripotency of human pluripotent stem cells
Source: Commun Biol. 2020 Sep 7;3:492. doi: 10.1038/s42003-020-01218-3 (PMC7476926; doi:10.1038/s42003-020-01218-3)
Supplement: Supplementary file 1 — Supplementary Information [file 42003_2020_1218_MOESM1_ESM.pdf]

Supplementary Figures

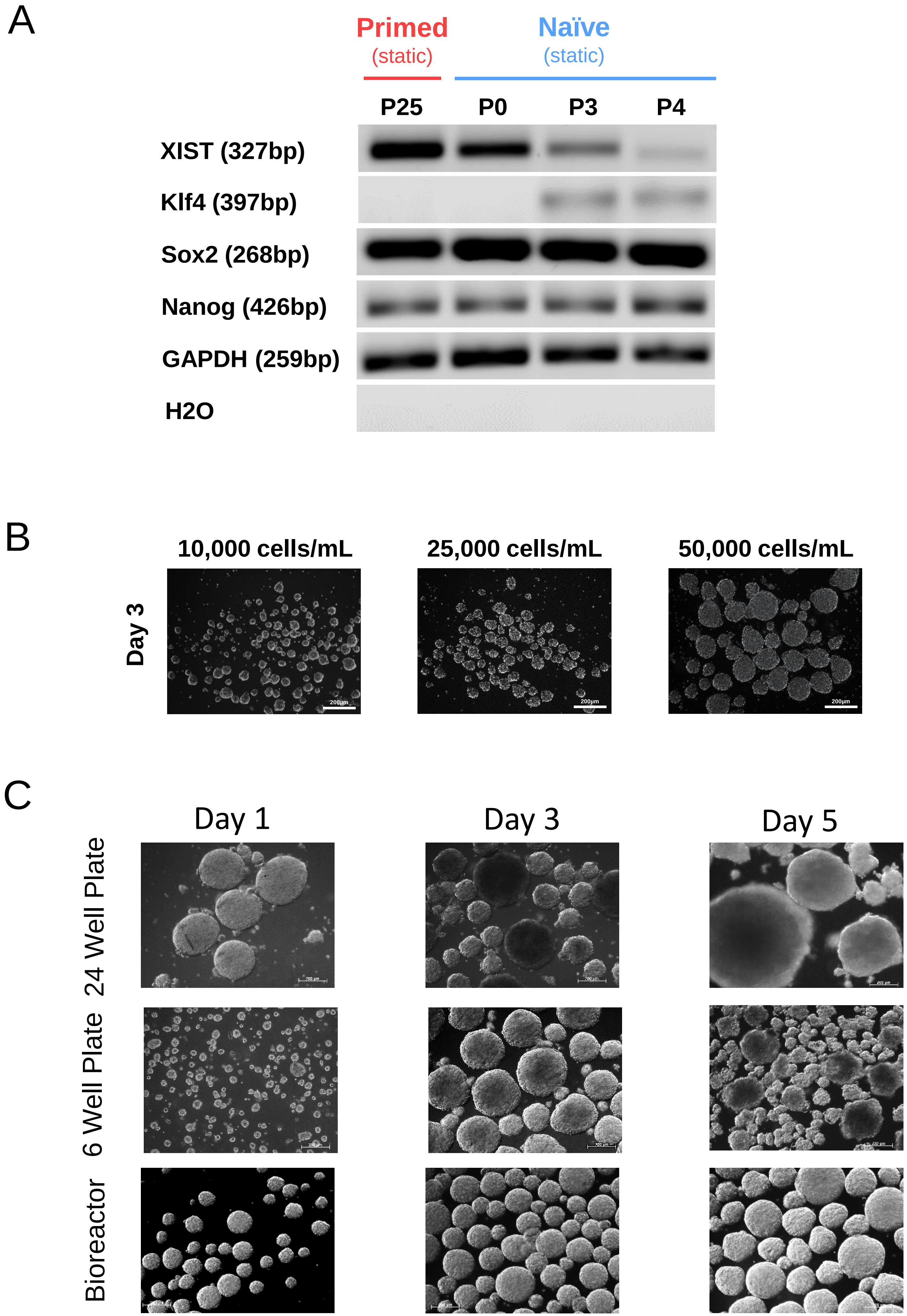
A
Primed
(static)
Naïve
(static)
P25
P0
P3
P4
XIST (327bp)
Klf4 (397bp)
Sox2 (268bp)
Nanog (426bp)
GAPDH (259bp)
H2O
B
10,000 cells/mL
25,000 cells/mL
50,000 cells/mL
Day 3
200μm
C
Day 1
Day 3
Day 5
24 Well Plate
6 Well Plate
Bioreactor

Supplementary Figure 1 (Related to Figures 1 & 2). a) Expression of pluripotency- and naïve-associated transcripts for naïve and primed H9 hPSCs in static culture using RT-PCR. Expression is shown for pluripotency (Sox2 & Nanog)- and naïve (XIST & KLF4)-related genes in naïve hPSCs (passages zero, three, and four after conversion) and primed H9 hPSCs during several passages in static cultures. GAPDH was used as internal standard. **b)** Naïve hPSC aggregates at different inoculation densities in stirred suspension bioreactor. The images show bioreactor-cultured, naïve H9 hPSC aggregates at day three post-inoculation tested at various seeding densities. Scale bars = 200 µM. **c)** Naïve hPSC aggregates at high seeding densities in shaken suspension well-plates, and low density in stirred suspension bioreactors. The images show naïve H1 hPSC aggregates cultured in 24 and 6-well shaken suspension plates at seeding densities of 1E5 and 2E5 cells/mL respectively, and at seeding density of 5E4 cells/mL in stirred suspension bioreactors for 5 days. Cultures underwent a single 60% media exchange on day 3.

A

	Naïve P1	Naïve P5	Primed
**Growth Rate (hr^{-1})**	0.0108±0.0025	0.0104±0.0009	0.0069±0.0005
**Doubling Time (hr)**	64.18±0.23	66.65±0.09	100.46±0.07
**Multiplication Ratio (Day 5)**	4.09±0.09	6.90±0.03	0.86±0.02

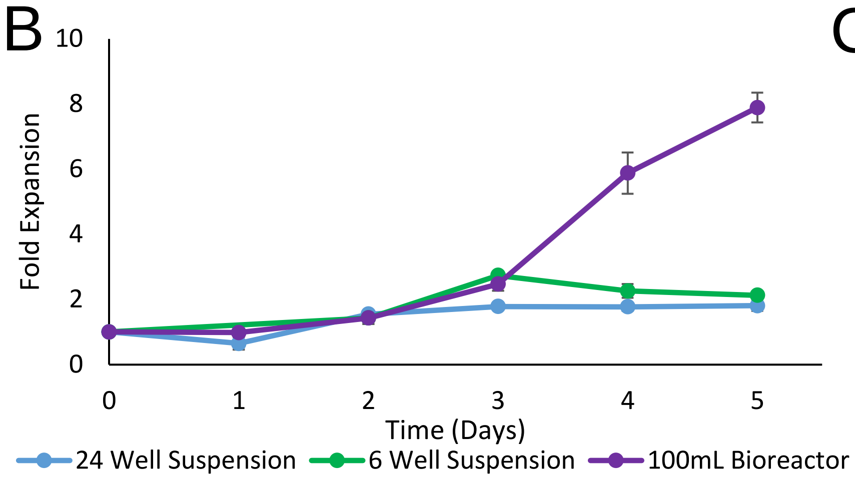

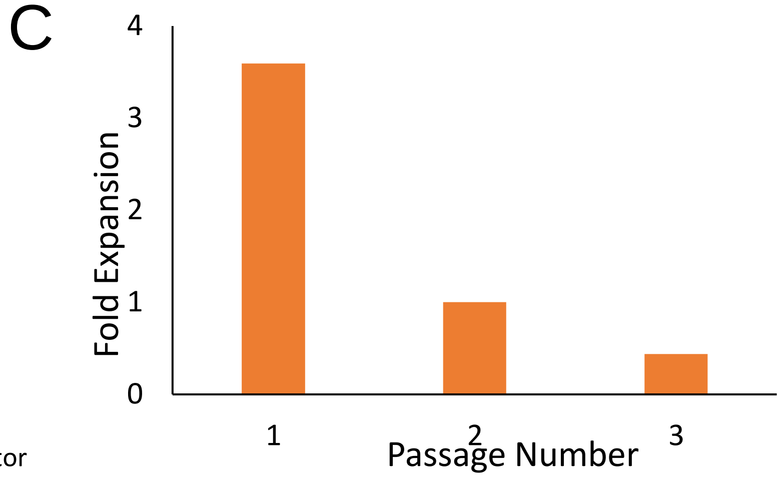

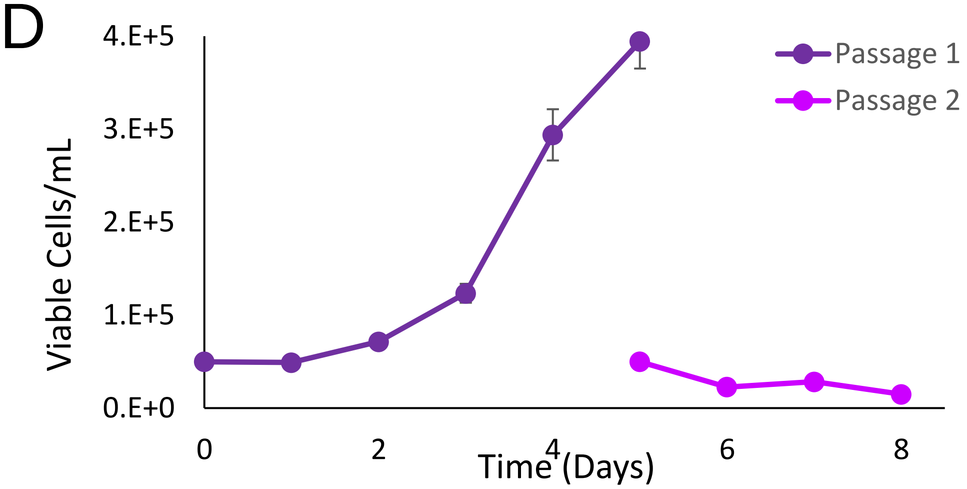

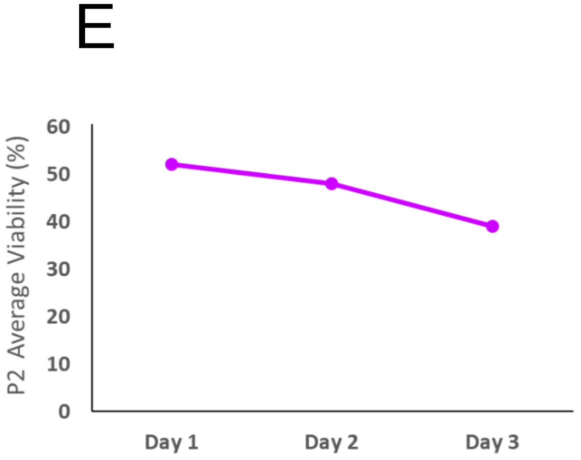

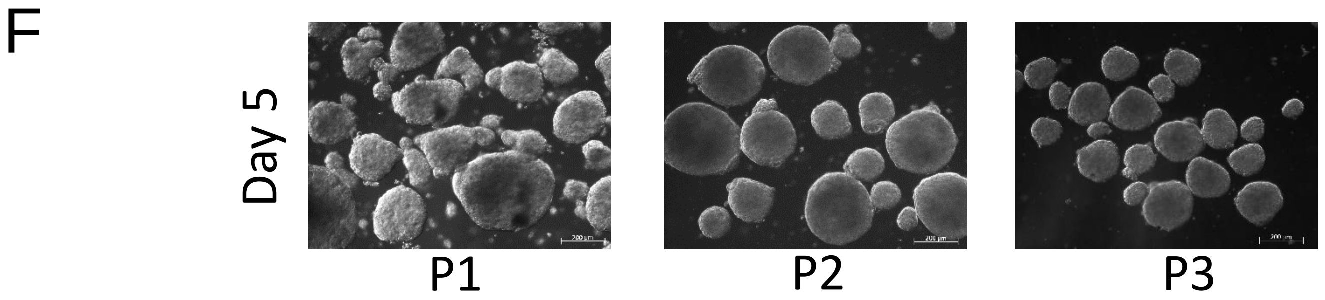

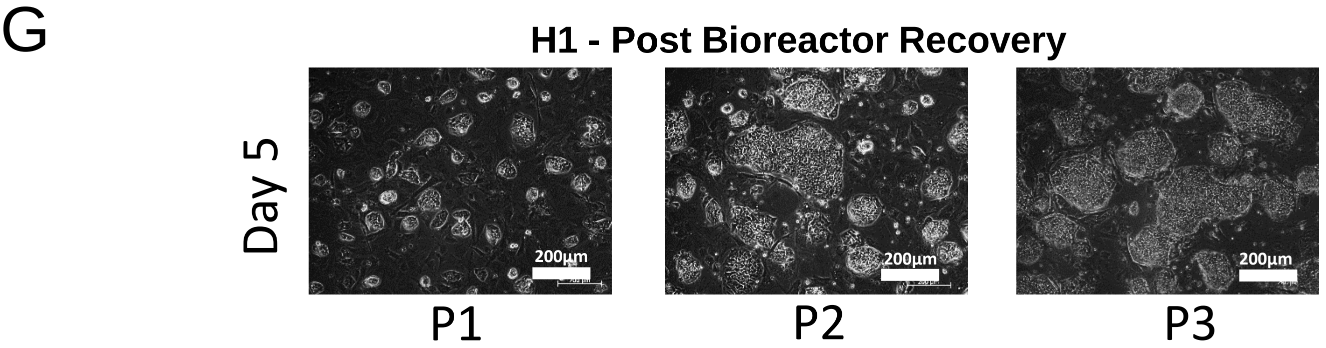

Supplementary Figure 2 (Related to Figure 2). a) Summary table of growth rate, exponential doubling time, and multiplication ratios of naïve (P1 and P5) and primed H9 hPSCs in stirred suspension bioreactor. The data presented are generated from inoculation density of 50,000 cells/mL. **b)** Fold expansion of naïve H1 hPSCs cultured in 24 and 6-well shaken suspension plates at densities of 1E5 and 2E5 cells/mL (high densities) for 5 days compared to the bioreactor culture seeded at 5E4 cells/mL (low density). Cultures underwent a single 60% media exchange on day 3. **c)** Serial passaging of naïve H1 hPSCs in 6-well shaken suspension plates (seeded at 2E5 cells/mL), passaged every 5 days. **d)** Serial passaging of naïve H1 hPSCs in 100 mL stirred suspension bioreactor (seeded at 50,000cells/mL), passaged every 5 days. The cultures underwent fed-batch condition (60% media change, 48h post-inoculation) for bioreactor culture. **e)** Average viabilities percentages of naïve hPSCs at first three days of passage two in 100 mL stirred suspension bioreactor (seeded at 50,000cells/mL). **f)** The images show naïve H1 hPSC aggregates following serial passaging, at day 5 post-inoculation. **g)** The images show naïve H1 hPSC aggregates recovered on static MEFs following serial passages in the bioreactor. Scale bars = 200 µM.

A

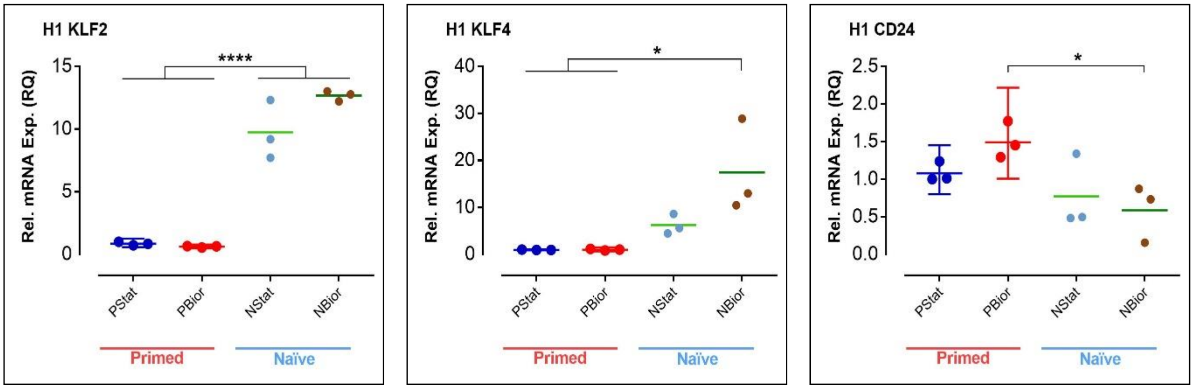
H1 KLF2
Rel. mRNA Exp. (RQ)
****
H1 KLF4
*
H1 CD24
PStat
PBior
NStat
NBior
Primed
Naïve

B

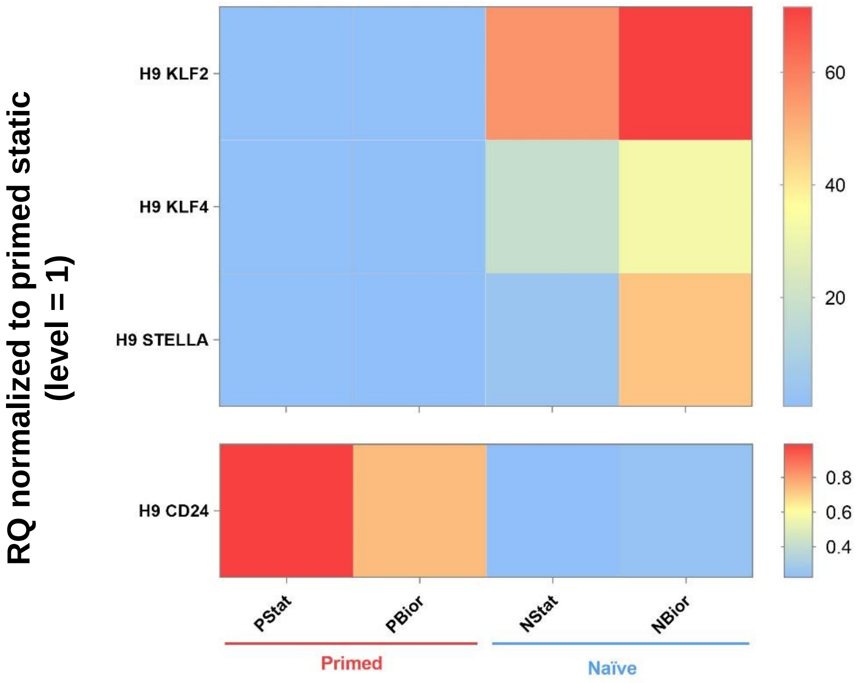
RQ normalized to primed static
(level = 1)
H9 KLF2
H9 KLF4
H9 STELLA
H9 CD24
60
40
20
0.8
0.6
0.4
PStat
PBior
NStat
NBior
Primed
Naïve

C

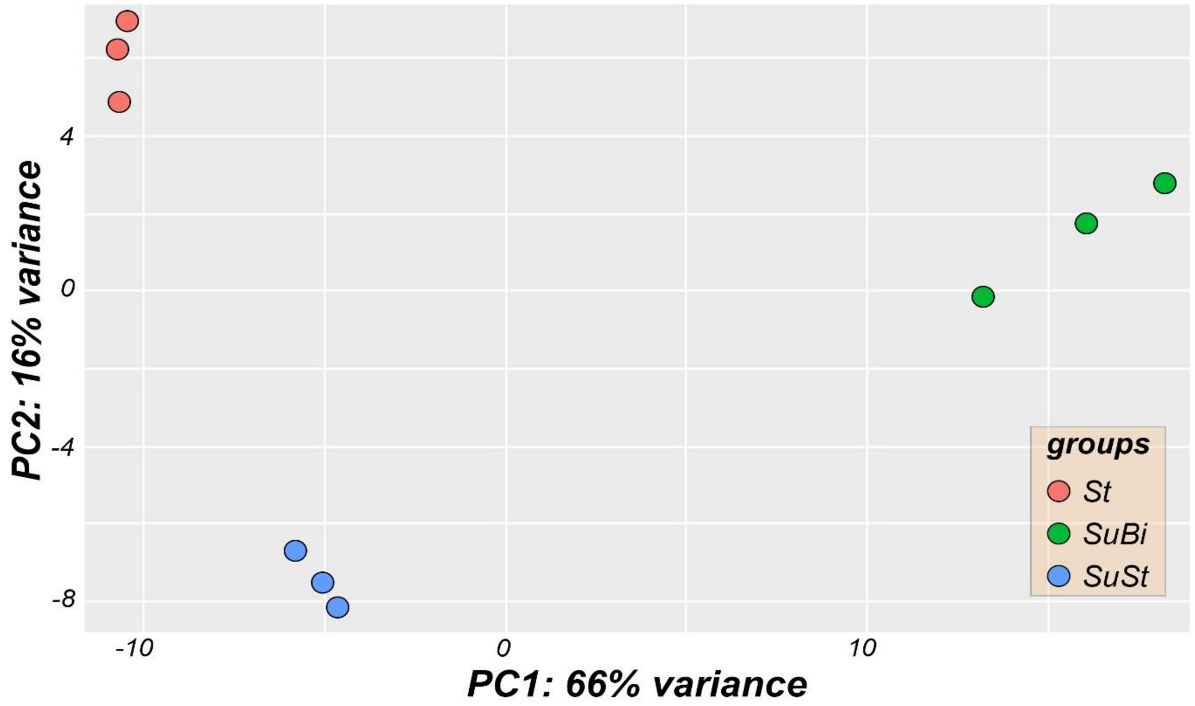
PC2: 16% variance
PC1: 66% variance
groups
St
SuBi
SuSt

Supplementary Figure 3 (Related to Figures 3 & 4). Gene expression and transcriptomic analyses of naïve and primed hPSCs. a) The dotplot Geometric Mean Linear depiction of naïve- (KLF2 & KLF4) and primed- (CD24) hPSC-associated gene expression levels for H1 hESC lines analyzed by RT-qPCR. The aliquots of statically-cultured cells for each naïve (P4) and primed hPSC sample were collected for gene expression analysis before inoculating them into stirred suspension bioreactors. Those cells were counted as “NStat” and “PStat”. The aggregates of day four post-inoculation were collected for each naïve and primed hPSC sample for gene expression analysis. Expression was quantified relative to the housekeeping gene GAPDH and was normalized to statically-cultured, primed hPSC (PStat) level (= 1). All the cultures underwent fed-batch condition (60% media change, 48h post-inoculation) for bioreactor cultures. The data presented are generated from inoculation density of 50,000 cells/mL. The data are represented as mean ± SEM (n = 3). **** = Adj-P < 0.0001, *** = Adj-P = 0.0002 & 0.0004, ** = Adj-P = 0.0021, * = Adj-P = 0.0291 using GraphPad Prism. Adj-P = Adjusted P-value. RQ = relative quantification. PStat = primed hPSCs cultured under static condition. PBior = primed hPSCs cultured under stirred suspension bioreactor condition. NStat = naïve hPSCs cultured under static condition. NBior = naïve hPSCs cultured under stirred suspension bioreactor condition. **b)** Heatmap of relative naïve- and primed- H9 hPSC specific gene expression levels normalized to statically-cultured, primed hPSC level (= 1). Red and blue colours represent upregulated and downregulated genes, respectively. RQ = relative quantification. **c)** A principal component analysis (PCA) of naïve H9 hPSCs cultured under static, static suspension, and stirred suspension culture. The first component of PCA discriminates between the cells from static suspension and stirred suspension culture (PC1, 66% variance). The second component discriminates between the cells from static suspension and static culture (PC2, 16% variance). The data are representative of three replicates.

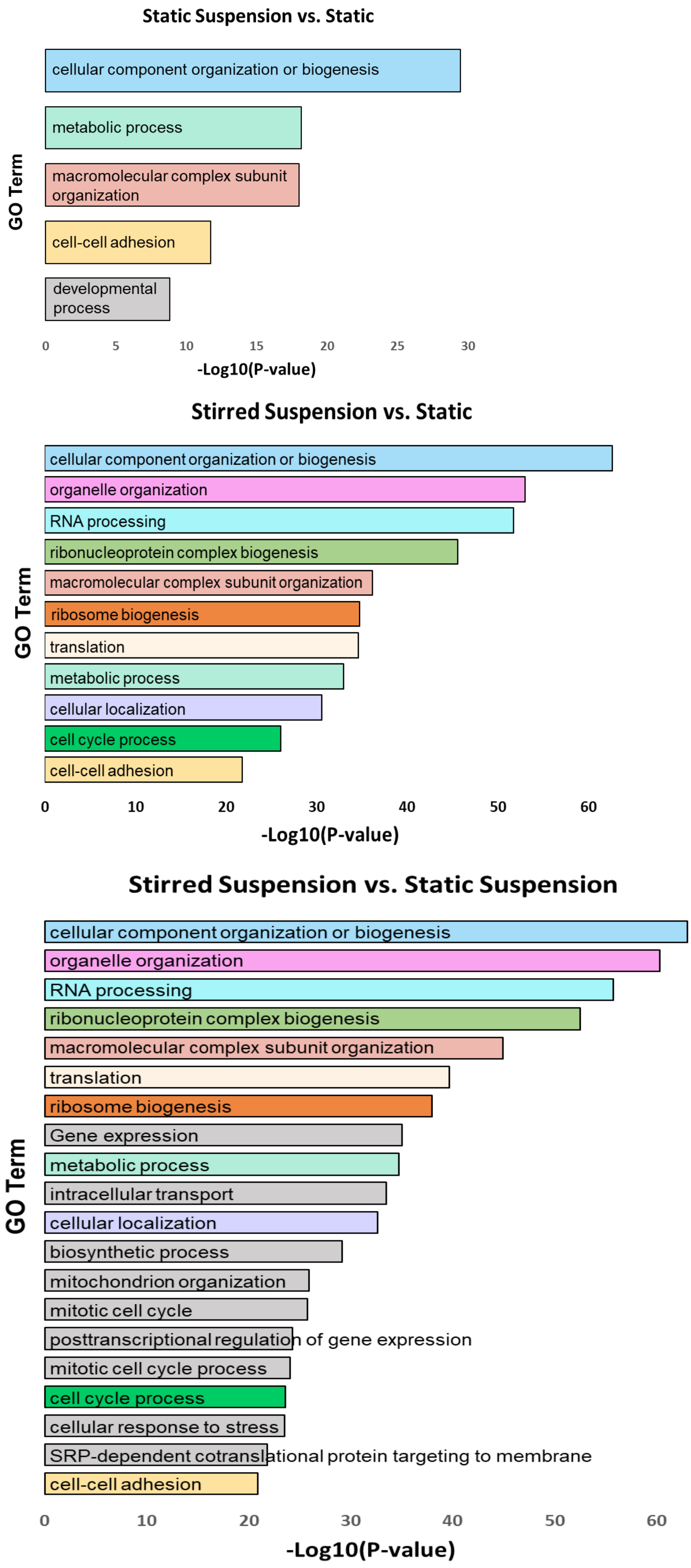
Static Suspension vs. Static
cellular component organization or biogenesis
metabolic process
macromolecular complex subunit organization
cell-cell adhesion
developmental process
GO Term
0
5
10
15
20
25
30
-Log10(P-value)
Stirred Suspension vs. Static
cellular component organization or biogenesis
organelle organization
RNA processing
ribonucleoprotein complex biogenesis
macromolecular complex subunit organization
ribosome biogenesis
translation
metabolic process
cellular localization
cell cycle process
cell-cell adhesion
GO Term
0
10
20
30
40
50
60
-Log10(P-value)
Stirred Suspension vs. Static Suspension
cellular component organization or biogenesis
organelle organization
RNA processing
ribonucleoprotein complex biogenesis
macromolecular complex subunit organization
translation
ribosome biogenesis
Gene expression
metabolic process
intracellular transport
cellular localization
biosynthetic process
mitochondrion organization
mitotic cell cycle
posttranscriptional regulation of gene expression
mitotic cell cycle process
cell cycle process
cellular response to stress
SRP-dependent cotranslational protein targeting to membrane
cell-cell adhesion
GO Term
0
10
20
30
40
50
60
-Log10(P-value)

Supplementary Figure 4 (Related to Figures 3 & 4). Gene ontology (GO) analysis of transcriptomic comparisons for naïve hPSCs cultured under static, static suspension and stirred suspension culture. Top enriched GO terms significantly enriched in naïve hPSCs cultured in **a)** static suspension vs. static culture condition, **b)** stirred suspension vs. static culture condition, and **c)** stirred suspension vs. static suspension culture condition are shown. The GO terms were selected from biological process (BP) category generated using DAVID Bioinformatics Resources 6.8. The values are based on –log10(P-value). The input gene dataset to DAVID was differentially expressed genes from each pair-wise comparison. Grey bars represent unique GO terms in the pairwise comparisons, whereas shared terms across comparisons are indicated by uniquely colored bars. The data are representative of three replicates.

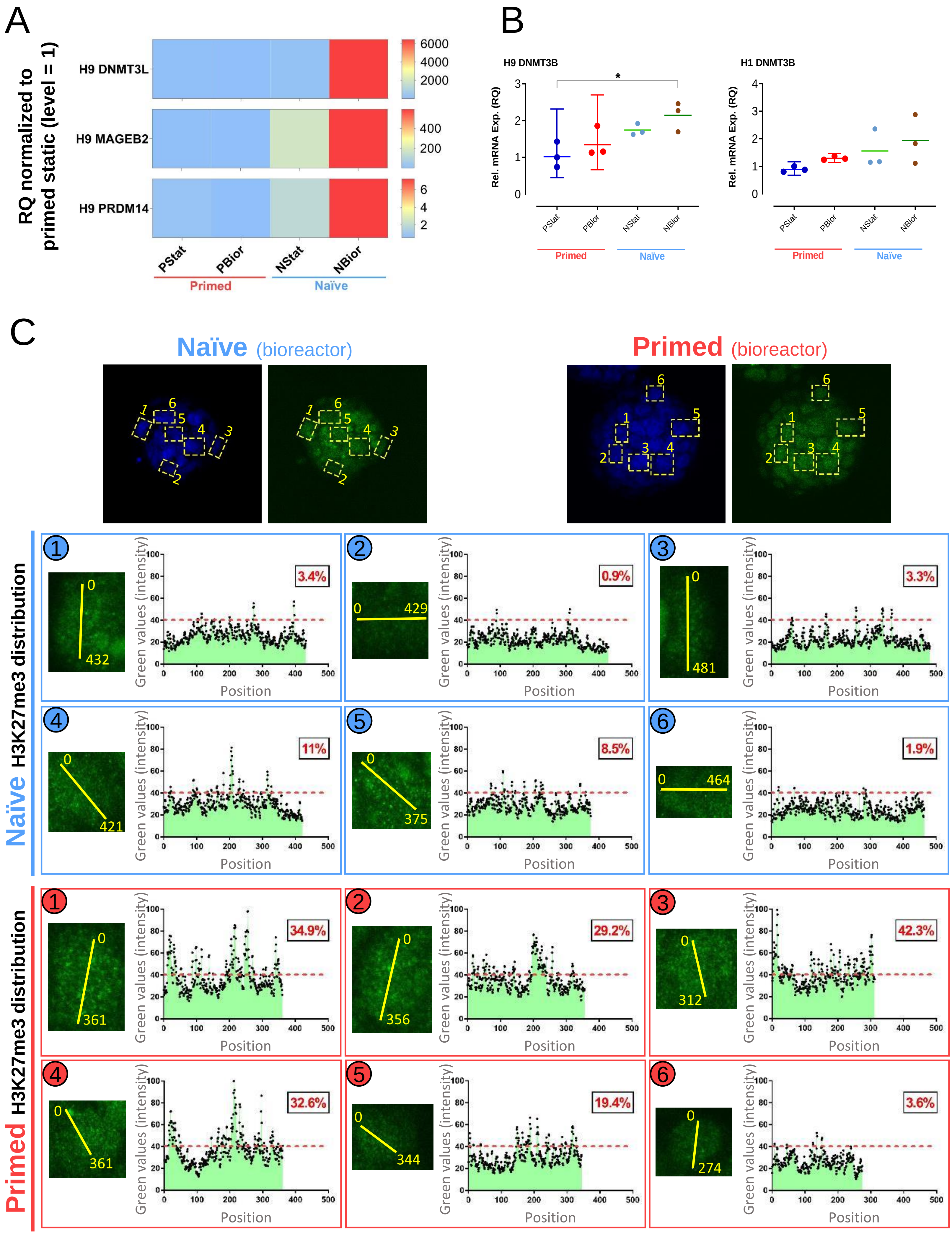

A
RQ normalized to primed static (level = 1)
H9 DNMT3L
H9 MAGEB2
H9 PRDM14
PStat
PBior
NStat
NBior
Primed
Naïve
B
H9 DNMT3B
H1 DNMT3B
Rel. mRNA Exp. (RQ)
C
Naïve (bioreactor)
Primed (bioreactor)
Naïve H3K27me3 distribution
Primed H3K27me3 distribution
Green values (intensity)
Position
3.4%
0.9%
3.3%
11%
8.5%
1.9%
34.9%
29.2%
42.3%
32.6%
19.4%
3.6%

Supplementary Figure 5 (Related to Figure 5). Epigenetic characterization of naïve and primed hPSCs. a) Heatmap of relative epigenetic regulator-related gene expression levels in naïve and primed H9 hPSCs, normalized to the statically-cultured, primed hPSC level (= 1), with red and blue colours representing upregulated and downregulated genes, respectively. RQ = relative quantification. **b)** Expression of *de novo* DNA methyltransferase in naïve and primed hPSCs. The dotplot Geometric Mean Linear depiction of DNMT3B epigenetic regulator transcript level for H1 and H9 hESC lines analyzed by RT-qPCR. The aliquots of statically-cultured cells for each naïve (P4) and primed hPSC sample were collected for gene expression analysis before inoculating them into stirred suspension bioreactors. Those cells were counted as "NStat" and "PStat". The aggregates of day four post-inoculation were collected for each naïve and primed hPSC sample for gene expression analysis. Expression was quantified relative to the housekeeping gene GAPDH and was normalized to statically-cultured primed hPSC (PStat) level (= 1). All the cultures underwent fed-batch condition (60% media change, 48h post-inoculation) for bioreactor cultures. The data presented are generated from inoculation density of 50,000 cells/mL. The data are represented as mean ± SEM (n = 3). * = Adj-P = 0.0199 using GraphPad Prism. Adj-P = Adjusted P-value. RQ = relative quantification. PStat = primed hPSCs cultured under static condition. PBior = primed hPSCs cultured under stirred suspension bioreactor condition. NStat = naïve hPSCs cultured under static condition. NBior = naïve hPSCs cultured under stirred suspension bioreactor condition. **c)** Intensity and distribution of H3K27me3 foci in the nuclei of bioreactor-cultured, naïve and primed H9 hPSC aggregates. Representative confocal images were obtained after whole-mount immunofluorescence for H3K27me3 on day four post-inoculated naïve and primed hPSC aggregates. Alexa Fluor 488 was used as a secondary antibody. Scale bars = 10 µm. Intensity and distribution of H3K27me3 foci were analyzed by Image J using Plot Profile analysis. The enlarged images of dashed boxes from six selected nuclei per naïve and primed hPSC aggregates are shown in blue (naïve) and red (primed) boxes under the aggregate's images. A symmetric midline was applied in naïve and primed hPSC aggregate nuclei (within enlarged images) using Plot Profile analysis in Image J to measure H3K27me3 intensity and distribution foci along the indicated midline. 432, 429, 481, 421, 375, and 464 foci for naïve hPSC nuclei and 361, 356, 312, 361, 344, and 274 foci for primed hPSC nuclei were analyzed along the midline. The H3K27me3 distribution graphs show the intensities of foci along the midline within the enlarged images. The x-axis represents the position of analyzed foci along the midline and the y-axis reflects the intensity value of the analyzed foci along the midline. The indicated numbers (%) within the black boxes show the percentage of dots which are located above the indicated arbitrary red line (the percentage of dots located above 40).

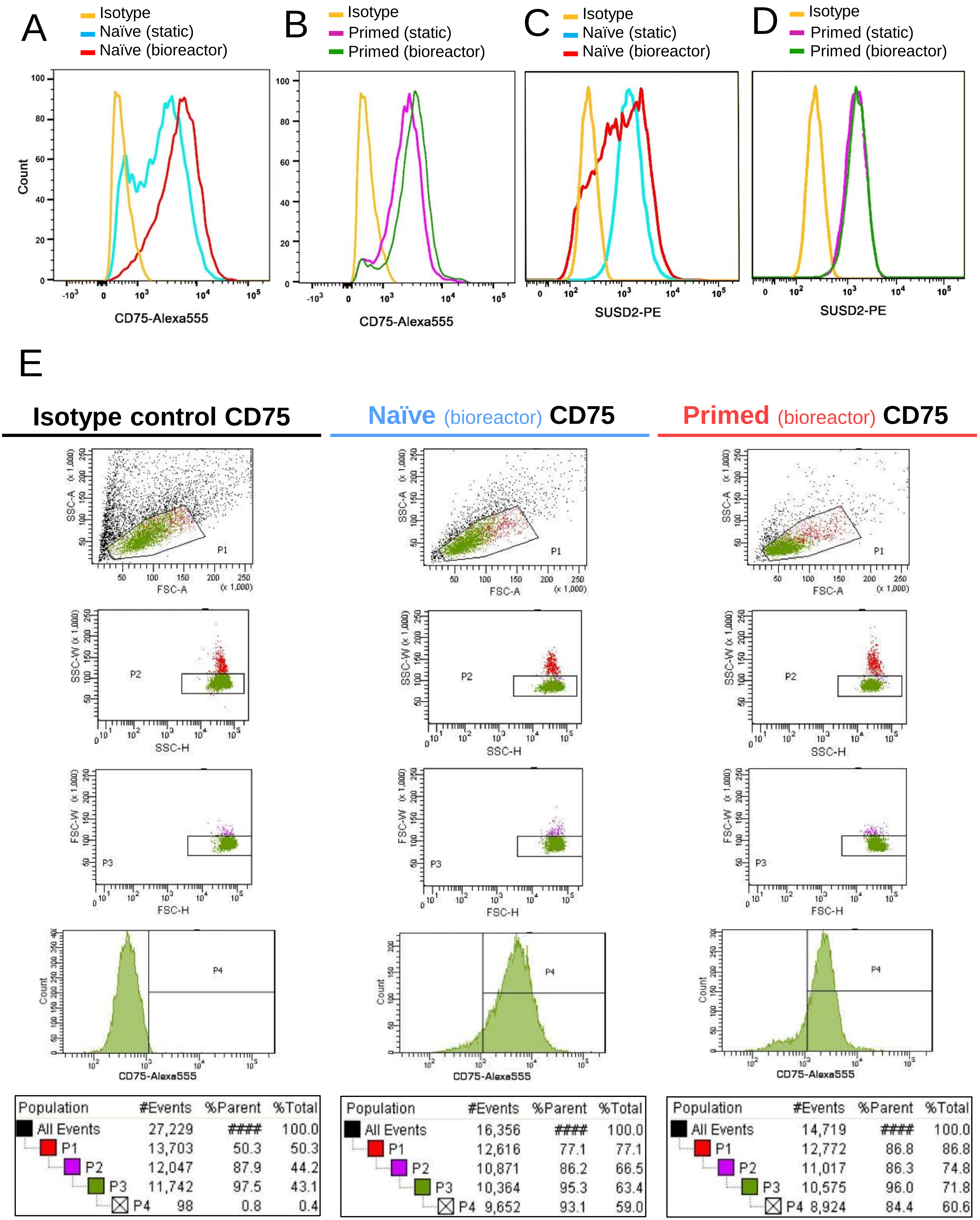
A
Isotype
Naïve (static)
Naïve (bioreactor)
Count
CD75-Alexa555
B
Isotype
Primed (static)
Primed (bioreactor)
CD75-Alexa555
C
Isotype
Naïve (static)
Naïve (bioreactor)
SUSD2-PE
D
Isotype
Primed (static)
Primed (bioreactor)
SUSD2-PE
E
Isotype control CD75
Naïve (bioreactor) CD75
Primed (bioreactor) CD75
SSC-A (x 1,000)
FSC-A (x 1,000)
P1
SSC-W (x 1,000)
SSC-H
P2
FSC-W (x 1,000)
FSC-H
P3
Count
CD75-Alexa555
P4
Population #Events %Parent %Total
All Events 27,229 #### 100.0
P1 13,703 50.3 50.3
P2 12,047 87.9 44.2
P3 11,742 97.5 43.1
P4 98 0.8 0.4
Population #Events %Parent %Total
All Events 16,356 #### 100.0
P1 12,616 77.1 77.1
P2 10,871 86.2 66.5
P3 10,364 95.3 63.4
P4 9,652 93.1 59.0
Population #Events %Parent %Total
All Events 14,719 #### 100.0
P1 12,772 86.8 86.8
P2 11,017 86.3 74.8
P3 10,575 96.0 71.8
P4 8,924 84.4 60.6

Supplementary Figure 6 (Related to Figure 7). Expression and gating strategy of naïve pluripotency cell surface protein markers in bioreactor-cultured, naïve and primed hPSCs and their statically-cultured counterparts. Histogram of flow cytometry analysis for CD75 cell surface protein marker in **a)** static- and bioreactor-cultured, naïve H9 hPSCs, **b)** static- and bioreactor-cultured, primed H9 hPSCs. Histogram of flow cytometry analysis for SUSD2 cell surface protein marker in **c)** static- and bioreactor-cultured, naïve H9 hPSCs, **d)** static- and bioreactor-cultured, primed H9 hPSCs. Gates were drawn based on an isotype control. The aliquots of static-cultured cells for each naïve (P4) and primed hPSC sample were collected for flowcytometry analysis before inoculating them into stirred suspension bioreactors. Aggregates of day four post-inoculation were used for flow cytometry analysis. All the cultures underwent fed-batch condition (60% media change, 48h post-inoculation) for bioreactor cultures. The data presented are generated from inoculation density of 50,000 cells/mL. **e)** Flow cytometry dot plots showing gating strategy for isotype control (left panel), bioreactor-cultured, naïve (middle panel) and primed (right panel) hPSCs. The first gate (P1) identifies the cell population based on the cell size (FSC) and complexity of the cells (SSC). The next two gates (P2 & P3) enable the discrimination of cells versus debris and excludes doublets and aggregates. The last gate demonstrates the expression level of the marker (CD75) on single cells in the gated population.

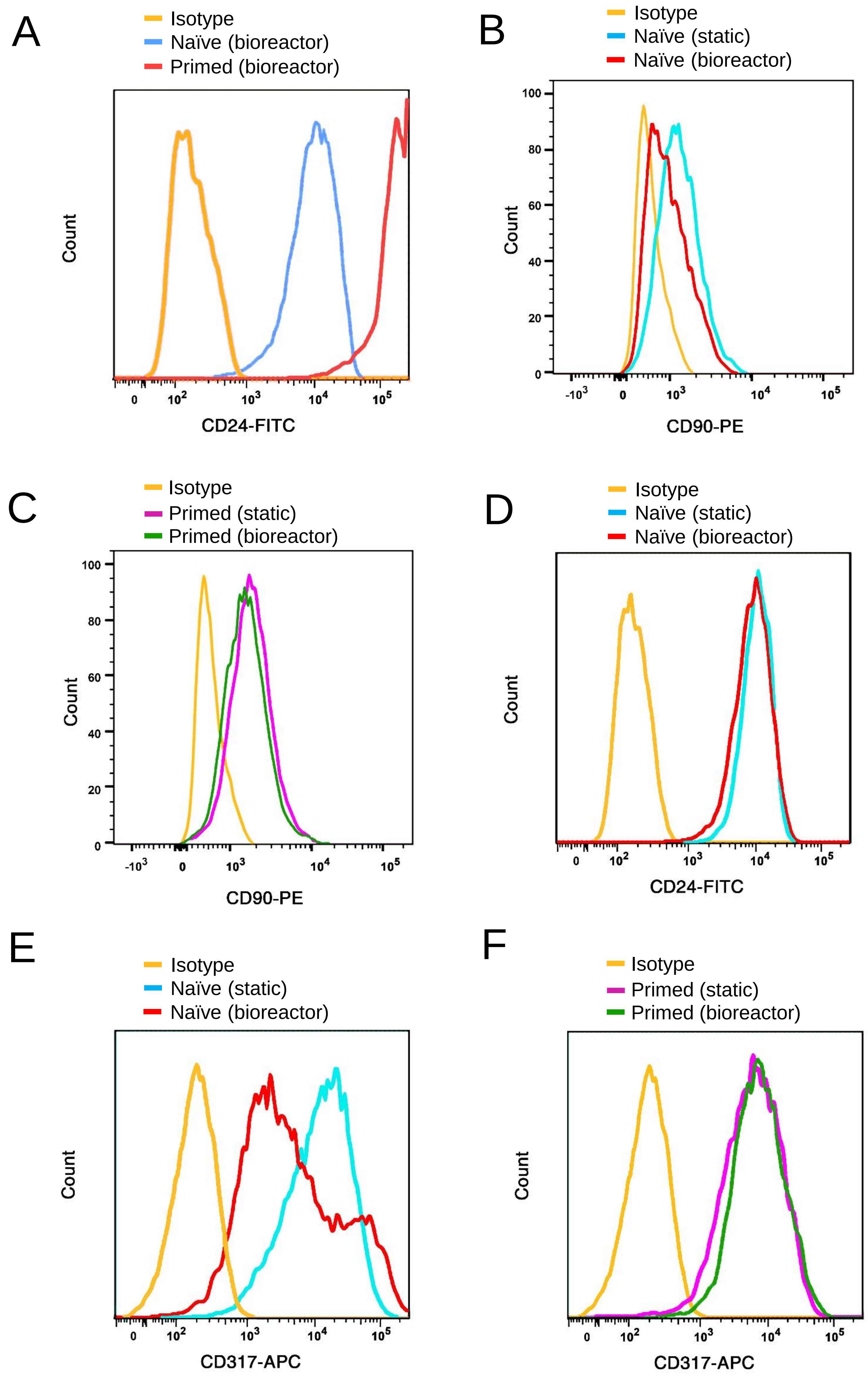

A
Isotype
Naïve (bioreactor)
Primed (bioreactor)
Count
CD24-FITC
B
Isotype
Naïve (static)
Naïve (bioreactor)
Count
CD90-PE
C
Isotype
Primed (static)
Primed (bioreactor)
Count
CD90-PE
D
Isotype
Naïve (static)
Naïve (bioreactor)
Count
CD24-FITC
E
Isotype
Naïve (static)
Naïve (bioreactor)
Count
CD317-APC
F
Isotype
Primed (static)
Primed (bioreactor)
Count
CD317-APC

Supplementary Figure 7 (Related to Figure 8). Expression of primed pluripotency cell surface protein markers in bioreactor-cultured, naïve and primed hPSCs and their statically-cultured counterparts. a) Histogram of flow cytometry analysis for CD24 cell surface protein marker in bioreactor-cultured, naïve and primed H1 hPSCs. Histogram of flow cytometry analysis for CD90 cell surface protein marker in **b)** static- and bioreactor-cultured, naïve H9 hPSCs, **c)** static- and bioreactor-cultured, primed H9 hPSCs. **d)** Histogram of flow cytometry analysis for CD24 cell surface protein marker in static- and bioreactor-cultured, naïve H1 hPSCs. Histogram of flow cytometry analysis for CD317 cell surface protein marker in **e)** static- and bioreactor-cultured, naïve H9 hPSCs, **f)** static- and bioreactor-cultured, primed H9 hPSCs. Gates were drawn based on an isotype control. The aliquots of static-cultured cells for each naïve (P4) and primed hPSC sample were collected for flowcytometry analysis before inoculating them into stirred suspension bioreactors. Aggregates of day four post-inoculation were used for flow cytometry analysis. All the cultures underwent fed-batch condition (60% media change, 48h post-inoculation) for bioreactor cultures. The data presented are generated from inoculation density of 50,000 cells/mL.

Isotype control CD90 | **Naïve** (bioreactor) **CD90** | **Primed** (bioreactor) **CD90**

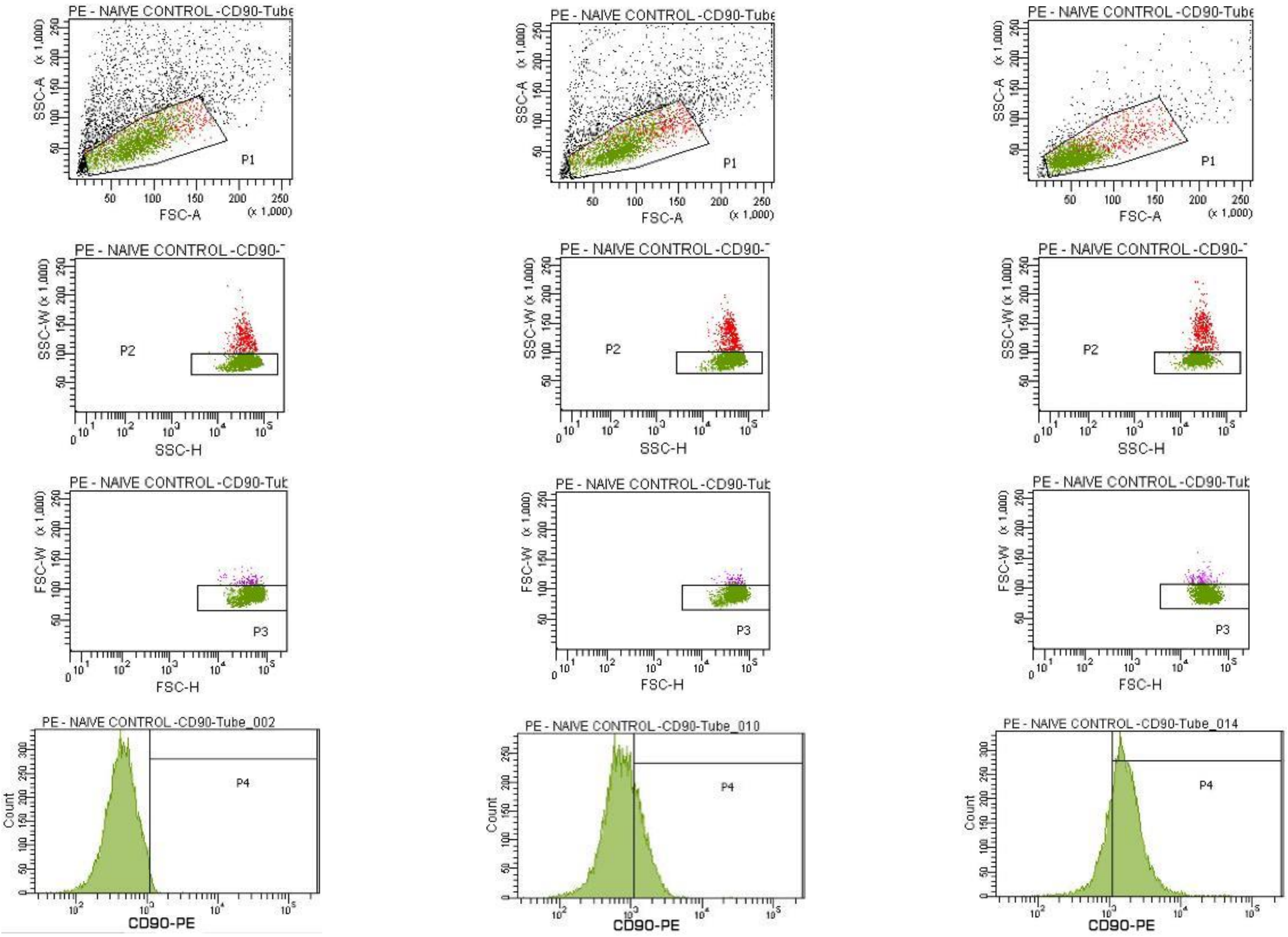

Population	#Events	%Parent	%Total
All Events	23,183	####	100.0
P1	12,617	54.4	54.4
P2	10,589	83.9	45.7
P3	10,131	95.7	43.7
P4	126	1.2	0.5

Population	#Events	%Parent	%Total
All Events	20,821	####	100.0
P1	12,796	61.5	61.5
P2	10,409	81.3	50.0
P3	10,074	96.8	48.4
P4	2,798	27.8	13.4

Population	#Events	%Parent	%Total
All Events	14,418	####	100.0
P1	13,033	90.4	90.4
P2	10,738	82.4	74.5
P3	10,197	95.0	70.7
P4	7,838	76.9	54.4

Supplementary Figure 8 (Related to Figure 8). Gating strategy for CD90 cell surface protein marker in bioreactor-cultured, naïve and primed hPSCs. Flow cytometry dot plots showing gating strategy for isotype control (left panel), bioreactor-cultured, naïve (middle panel) and primed (right panel) hPSCs. The first gate (P1) identifies the cell population based on the cell size (FSC) and complexity of the cells (SSC). The next two gates (P2 and P3) enable the discrimination of cells versus debris and exclude the doublets and aggregates. The last gate demonstrates expression level of the marker (CD90) on single cells in the gated population.

A

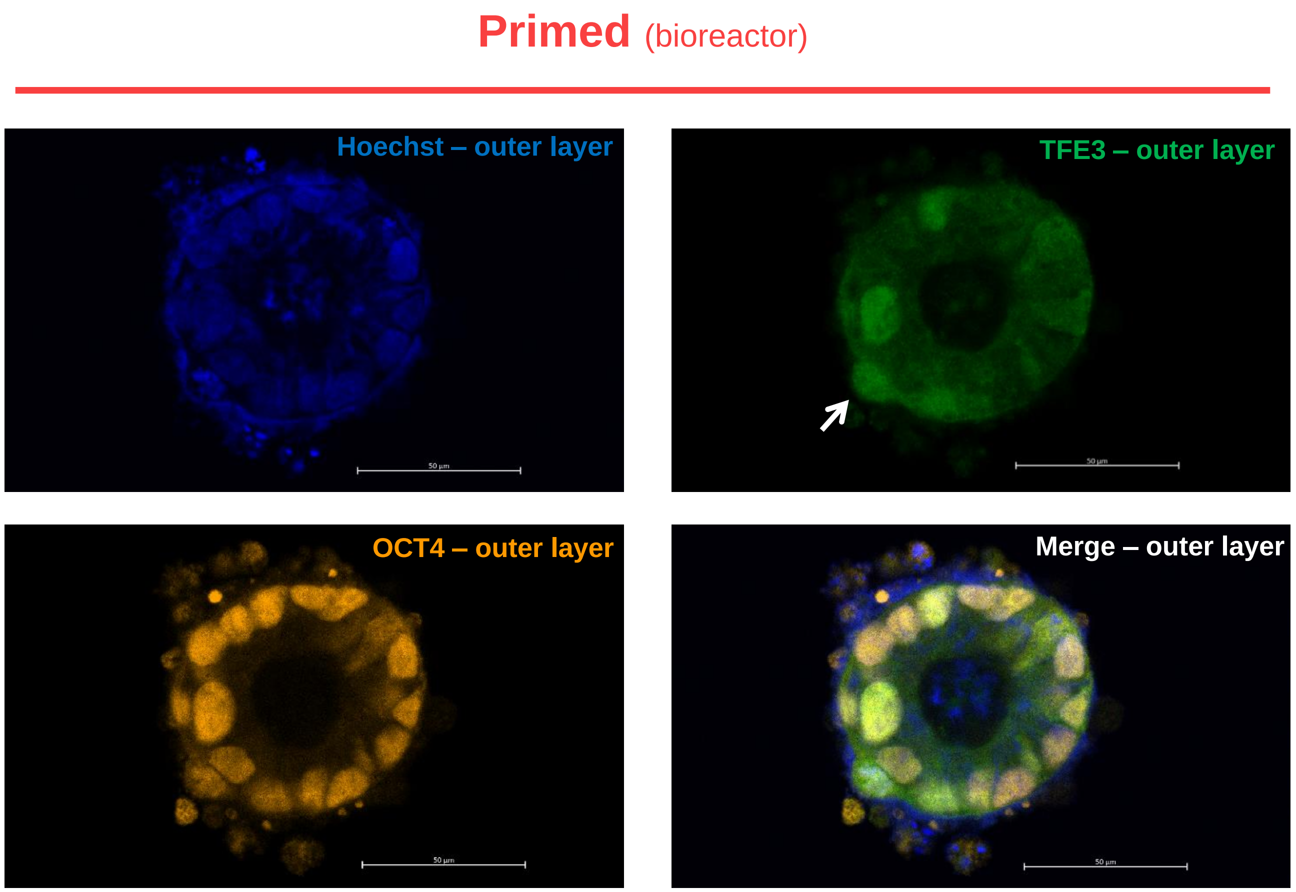

B

Cell type	Teratomas	Mesoderm	Endoderm	Ectoderm
**Naïve** (bioreactor)	3/3	+	+++	++
**Primed** (bioreactor)	3/3	+++	+	++

Supplementary Figure 9 (Related to Figures 9 & 10). Nuclear localization of TFE3 at the outer layer of bioreactor-cultured, primed hPSC aggregates, and overview table of teratoma results. **a)** Representative confocal images are shown of primed H9 hPSC aggregates double-immuno-stained for TFE3/OCT4. The arrowhead shows the nuclear localization of TFE3 at the outer layer of the aggregate. The aggregates on day four post-inoculation were used for whole-mount staining and confocal imaging. Scale bars = 50 µm. **b)** Overview table of results for teratoma formation from bioreactor-cultured, naïve and primed H9 hPSCs. Ratios indicate the number of teratomas formation over the number of animals injected for each cell type. The number of '**+**' signs indicates the degree of germ layer contribution.

A

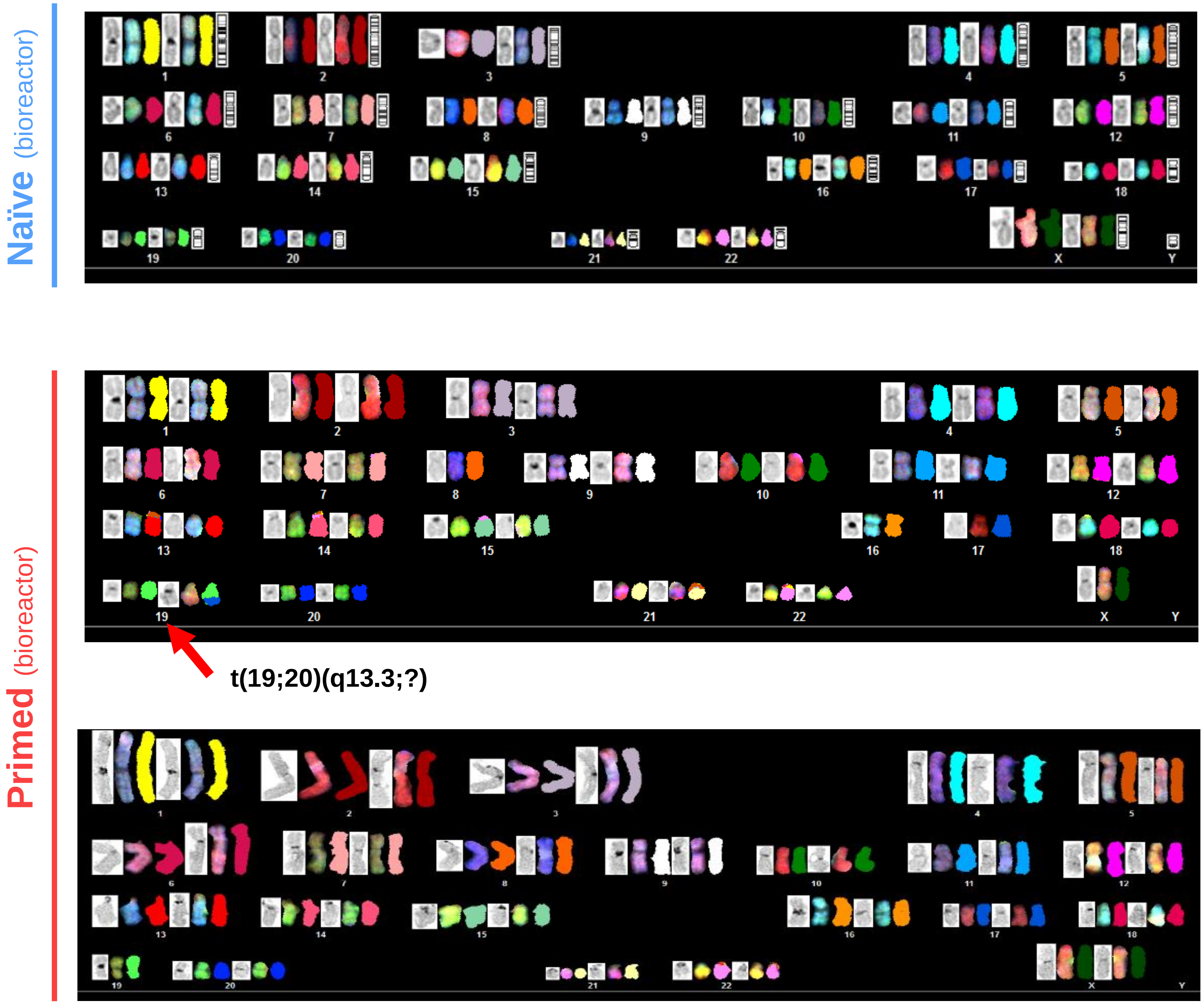

B

	Naïve (bioreactor)	Primed (bioreactor)
**Analyzed metaphases**	25	35
**Structural chromosomal aberrations**	0	Unbalanced nonreciprocal translocation between Chrs. 19 & 20 (in one metaphase)

Supplementary Figure 10 (Related to Figure 10). Chromosomal stability of bioreactor-cultured, naïve and primed hPSCs. a) Representative karyotypes of naïve and primed H9 hPSCs grown in bioreactor, analyzed via spectral karyotyping. Arrowhead in primed hPSC-related metaphase shows unbalanced nonreciprocal translocation between chromosomes 19 and 20 [t(19;20)(q13.3;?)] (one metaphase). **b)** Table summarizing the karyotype analyses (SKY) for structural chromosomal aberrations in naïve and primed H9 hPSCs cultured in bioreactor.

Supplementary Tables

Supplementary Table 1. Pairwise-transcriptomic comparison of naïve hPSCs cultured under static suspension and static culture. Relevant subset of enriched pathways in static suspension compared to static culture (selected as a control reference) generated from Ingenuity Pathway Analysis (IPA). Full data available in Supplementary Data 2.

Static Suspension vs. Static				
**Pathway**	Activated	Inactivated	No-expect	**Z-Score**
Mouse ESC Pluripotency	11 (30%)	25 (68%)	1 (3%)	**-2.302**
RhoA	9 (23%)	28 (72%)	2 (5%)	**-3.042**
Actin Cytoskeleton	18 (29%)	37 (60%)	7 (11%)	**-2.413**
AMPK	30 (54%)	12 (21%)	14 (25%)	**2.405**

Activated = genes expected to be upregulated (positive log2FC) if the pathway is activated according to IPA canonical pathway database.
Inactivated = genes expected to be downregulated (negative log2FC) if the pathway is activated according to IPA canonical pathway database
No-expect = no direction of change expectation is available in the IPA canonical pathway database

Z-score = an IPA algorithm used to identify enriched canonical pathways that are more active (positive z-score) or less active (negative z-score) according to IPA database and observed gene expression in RNA-seq dataset. Statistically measurement of the match between expected relationship direction (according to IPA database) and observed gene expression in RNA-seq dataset.

Filter criteria for pathway enrichment significance was expected direction of change Z-score ≥ 2 or ≤ -2, and –log10(Benjamini-Hochberg multiple testing corrected p-value of differential expression) ≥ 2.

Negative z-score = a hallmark of negative enrichment for a pathway, which implies “down-regulation”

Positive z-score = a hallmark of positive enrichment for a pathway, which implies “up-regulation”

Supplementary Table 2. Pairwise-transcriptomic comparison of naïve hPSCs cultured under stirred suspension and static culture. Relevant subset of enriched pathways in stirred suspension compared to static culture (selected as a control reference) generated from Ingenuity Pathway Analysis (IPA). Full data available in Supplementary Data 4.

Stirred Suspension vs. Static				
**Pathway**	Activated	Inactivated	No-expect	**Z-Score**
HIPPO	23 (57%)	14 (35%)	3 (8%)	**3.162**
Synaptogenesis	35 (28%)	5 (4%)	84 (68%)	**-4.4**
Stearate Biosynthesis	5 (21%)	18 (75%)	1 (4%)	**-2.654**
Integrin	17 (18%)	7 (7%)	70 (74%)	**-5.467**
CREB Signaling in Neurons	9 (10%)	39 (44%)	40 (45%)	**-3.305**
Ephrin Receptor	8 (10%)	14 (18%)	55 (71%)	**-5.356**
Endocannabinoid Developing Neuron	14 (26%)	5 (9%)	34 (64%)	**-2.747**
Phagocytosis in Macrophages & Monocytes	11 (25%)	1 (2%)	32 (73%)	**-3.166**

Activated = genes expected to be upregulated (positive log2FC) if the pathway is activated according to IPA canonical pathway database.
Inactivated = genes expected to be downregulated (negative log2FC) if the pathway is activated according to IPA canonical pathway database
No-expect = no direction of change expectation is available in the IPA canonical pathway database

Z-score = an IPA algorithm used to identify enriched canonical pathways that are more active (positive z-score) or less active (negative z-score) according to IPA database and observed gene expression in RNA-seq dataset. Statistically measurement of the match between expected relationship direction (according to IPA database) and observed gene expression in RNA-seq dataset.

Filter criteria for pathway enrichment significance was expected direction of change Z-score ≥ 2 or ≤ -2, and –log10(Benjamini-Hochberg multiple testing corrected p-value of differential expression) ≥ 2.

Negative z-score = a hallmark of negative enrichment for a pathway, which implies "down-regulation"

Positive z-score = a hallmark of positive enrichment for a pathway, which implies "up-regulation"

Supplementary Table 3. Pairwise-transcriptomic comparison of naïve hPSCs cultured under stirred suspension and static suspension culture. Relevant subset of enriched pathways in stirred suspension compared to static suspension culture (selected as a control reference) generated from Ingenuity Pathway Analysis (IPA). Full data available in Supplementary Data 6.

Stirred Suspension vs. Static Suspension				
**Pathway**	Activated	Inactivated	No-expect	**Z-Score**
HIPPO	19 (46%)	5 (12%)	17 (41%)	**2.186**
PTEN	42 (69%)	15 (25%)	4 (7%)	**3.457**
ERK-MAPK	27 (31%)	53 (62%)	6 (7%)	**-2.804**
Cholesterol Biosynthesis	1 (5%)	17 (89%)	1 (5%)	**-3.671**
Cardiac Hypertrophy	24 (24%)	63 (64%)	11 (11%)	**-3.94**
Integrin	18 (20%)	65 (71%)	8 (9%)	**-4.927**
CDK5	15 (31%)	31 (63%)	3 (6%)	**-2.286**
CREB Signaling in Neurons	11 (13%)	37 (44%)	37 (44%)	**-2.82**
Endocannabinoid Developing Neuron	9 (18%)	37 (73%)	5 (10%)	**-3.921**
Ephrin B Signaling	3 (8%)	24 (60%)	13 (32%)	**-3.32**
Ephrin Receptor	10 (12%)	60 (71%)	14 (17%)	**-5.455**
fMLP in Neutrophils	12 (23%)	36 (69%)	4 (8%)	**-3.328**
RhoA	16 (28%)	37 (65%)	4 (7%)	**-2.782**

Activated = genes expected to be upregulated (positive log2FC) if the pathway is activated according to IPA canonical pathway database.
Inactivated = genes expected to be downregulated (negative log2FC) if the pathway is activated according to IPA canonical pathway database
No-expect = no direction of change expectation is available in the IPA canonical pathway database

Z-score = an IPA algorithm used to identify enriched canonical pathways that are more active (positive z-score) or less active (negative z-score) according to IPA database and observed gene expression in RNA-seq dataset. Statistically measurement of the match between expected relationship direction (according to IPA database) and observed gene expression in RN-Aseq dataset.

Filter criteria for pathway enrichment significance was expected direction of change Z-score ≥ 2 or ≤ -2, and –log10(Benjamini-Hochberg multiple testing corrected p-value of differential expression) ≥ 2.

Negative z-score = a hallmark of negative enrichment for a pathway, which implies “down-regulation”

Positive z-score = a hallmark of positive enrichment for a pathway, which implies “up-regulation”
