# Supplementary material for: Stirred suspension bioreactors maintain naïve pluripotency of human pluripotent stem cells
Source: Commun Biol. 2020 Sep 7;3:492. doi: 10.1038/s42003-020-01218-3 (PMC7476926; doi:10.1038/s42003-020-01218-3)
Supplement: Supplementary file 20 — Description of Additional Supplementary Files [file 42003_2020_1218_MOESM20_ESM.pdf]

Description of Additional Supplementary Files

Supplementary Data 1 (.xlsx) Differentially expressed transcripts in suspension static vs. static culture condition

Supplementary Data 2 (.xlsx) Enriched canonical pathways in suspension static vs. static culture condition

Supplementary Data 3 (.xlsx) Differentially expressed transcripts in stirred suspension vs. static culture condition

Supplementary Data 4 (.xlsx) Enriched canonical pathways in stirred suspension vs. static culture condition

Supplementary Data 5 (.xlsx) Differentially expressed transcripts in stirred suspension vs. static suspension culture condition

Supplementary Data 6 (.xlsx) Enriched canonical pathways in stirred suspension vs. static suspension culture condition

Supplementary Data 7 (.xlsx) Enriched GO terms in static suspension vs. static culture condition

Supplementary Data 8 (.xlsx) Enriched GO terms in stirred suspension vs. static culture condition

Supplementary Data 9 (.xlsx) Enriched GO terms in stirred suspension vs. static suspension culture condition

Supplementary Data 10 (.xlsx) Processed Metabolomics Data

Supplementary Data 11 (.xlsx) TaqMan probes for RT-qPCR SYBR Green primer sequences for RT-qPCR RT-PCR primer sequences

Supplementary Movie 1 (.mp4) H3K27me3 expression foci in naïve aggregate

Supplementary Movie 2 (.mp4) H3K27me3 expression foci in primed aggregate

Supplementary Movie 3 (.mp4) TFE3 and OCT4 co-expression in naïve aggregate

Supplementary Movie 4 (.mp4) KLF4 and STELLA co-expression in naïve aggregate

Supplementary Movie 5 (.mp4) TFE3 and OCT4 co-expression in primed aggregate

Supplementary Movie 6 (.mp4) KLF4 and STELLA co-expression in primed aggregate

Supplementary Movie 7 (.avi) Beating cardiomyocytes differentiated from bioreactor-cultured naïve aggregates
