# Supplementary material for: Stirred suspension bioreactors maintain naïve pluripotency of human pluripotent stem cells
Source: Commun Biol. 2020 Sep 7;3:492. doi: 10.1038/s42003-020-01218-3 (PMC7476926; doi:10.1038/s42003-020-01218-3)
Supplement: Supplementary file 21 — Reporting Summary [file 42003_2020_1218_MOESM21_ESM.pdf]

nature research

Corresponding author(s): Derrick E. Rancourt

Last updated by author(s): Jul 17, 2020

Reporting Summary

Nature Research wishes to improve the reproducibility of the work that we publish. This form provides structure for consistency and transparency in reporting. For further information on Nature Research policies, see Authors & Referees and the Editorial Policy Checklist.

Statistics

For all statistical analyses, confirm that the following items are present in the figure legend, table legend, main text, or Methods section.

n/a	Confirmed	
☐	☒	The exact sample size (*n*) for each experimental group/condition, given as a discrete number and unit of measurement
☐	☒	A statement on whether measurements were taken from distinct samples or whether the same sample was measured repeatedly
☐	☒	The statistical test(s) used AND whether they are one- or two-sided<br>*Only common tests should be described solely by name; describe more complex techniques in the Methods section.*
☒	☐	A description of all covariates tested
☐	☒	A description of any assumptions or corrections, such as tests of normality and adjustment for multiple comparisons
☐	☒	A full description of the statistical parameters including central tendency (e.g. means) or other basic estimates (e.g. regression coefficient) AND variation (e.g. standard deviation) or associated estimates of uncertainty (e.g. confidence intervals)
☐	☒	For null hypothesis testing, the test statistic (e.g. *F*, *t*, *r*) with confidence intervals, effect sizes, degrees of freedom and *P* value noted<br>*Give P values as exact values whenever suitable.*
☒	☐	For Bayesian analysis, information on the choice of priors and Markov chain Monte Carlo settings
☒	☐	For hierarchical and complex designs, identification of the appropriate level for tests and full reporting of outcomes
☒	☐	Estimates of effect sizes (e.g. Cohen's *d*, Pearson's *r*), indicating how they were calculated

Our web collection on statistics for biologists contains articles on many of the points above.

Software and code

Policy information about availability of computer code

Data collection	AxioVision Rel. 4.8 was used to determine average aggregate size (diameters) of bioreactor-cultured naive and primed hPSC aggregates.<br>GraphPad Prism was used to analyze RT-qPCR results.<br>FastQC v0.11.5 was used to check the quality of raw sequenced reads in RNAseq analysis.<br>Kallisto 0.42.4 was used to pseudoalign RNA-seq reads to the human Ensembl RefSeq transcript database; GRCh38 (latest Ensembl human transcript reference).<br>DESeq (an R/Bioconductor package) was used for differential gene expression using static/static suspension/stirred suspension bioreactor as the explanatory variable.<br>Ingenuity Pathway Analysis (IPA) was used to identify enriched canonical pathways.<br>DAVID Bioinformatics Resources v6.8. was used for gene ontology analysis of transcriptomic data.<br>El-MAVEN was used for processing of metabolomics data.<br>R and MATLAB were used for statistical analysis and visual representation of metabolomics data.<br>FlowJo V10.1.3 was used for generation of histogram of flow cytometry analysis.<br>Zen Black (Carl Zeiss Microscopy) was used for Z-stack imaging.<br>Zen Blue (Carl Zeiss Microscopy) was used for image processing, generating three-dimensional view videos, and generating Z-stack videos.<br>Image J was used to analyze intensity and distribution of H3Kme3 foci in the nuclei of bioreactor-cultured naive and primed hPSC aggregates.
Data analysis	Plot Profile analysis was used to determine the intensity and distribution of H3Kme3 foci through Image J software.<br>Statistical analysis was done using GraphPad Prism.

For manuscripts utilizing custom algorithms or software that are central to the research but not yet described in published literature, software must be made available to editors/reviewers. We strongly encourage code deposition in a community repository (e.g. GitHub). See the Nature Research guidelines for submitting code & software for further information.

Data

Policy information about availability of data

All manuscripts must include a data availability statement. This statement should provide the following information, where applicable:

- Accession codes, unique identifiers, or web links for publicly available datasets
- A list of figures that have associated raw data
- A description of any restrictions on data availability

The authors declare that all data supporting the findings of this study are available within the article and its supplementary information files or from the corresponding author upon reasonable request. For RNA-seq data, the raw fastq files and raw count table containing the number of transcripts for each sample are available in GEO under accession number GSE144656.

Field-specific reporting

Please select the one below that is the best fit for your research. If you are not sure, read the appropriate sections before making your selection.

☒ Life sciences ☐ Behavioural & social sciences ☐ Ecological, evolutionary & environmental sciences

For a reference copy of the document with all sections, see nature.com/documents/nr-reporting-summary-flat.pdf

Life sciences study design

All studies must disclose on these points even when the disclosure is negative.

Sample size

Exact n values and statistical analysis for each experiment are defined and described in the main text, figure legends, and statistics at the end of "Methods" section.

We chose 4 biological replicates to detect growth kinetics of naive and primed hPSCs. Based on our previous works on culture of human and mouse pluripotent stem cells in stirred suspension bioreactor, having 4 biological replicates appropriately can determine growth kinetics of cells within stirred suspension bioreactors.

We chose 3 biological replicates for the following experiments in the revised manuscript: 1) Serial passaging of naive hPSCs, 2) RT-qPCR, 3) RNA-seq, 4) Metabolomics, 5) Whole-mount immunostaining and confocal imaging, 6) teratoma formation assay, and 7) In vitro differentiation.

For flow cytometry we used 2 biological replicates for some markers (CD75 & CD90), and 3 biological replicates for the rest of the markers in the revised manuscript.

Data exclusions

We applied serial passaging of naive hPSCs in 10 mL stirred suspension bioreactors in the revised manuscript, but we did not include the related results in the manuscript.

We performed RT-qPCR analysis several times to optimize the amount and concentration of cDNA samples as well as testing primer efficiencies. Those data were not included in the manuscript.

We performed RNAs-eq analysis for primed hPSCs cultured in stirred suspension bioreactor, however, we did not include it in the revised manuscript as it was 1 biological replicate, and was more exploratory.

Replication

The biological replicates which are included in the manuscript are reproducible.

Randomization

We allocated experimental groups to statically- and bioreactor- cultured naive and primed hPSCs to elucidate the influence of bioreactor environment on naive pluripotency.

Blinding

The allocation groups, data collection and analysis were blinded.

Reporting for specific materials, systems and methods

We require information from authors about some types of materials, experimental systems and methods used in many studies. Here, indicate whether each material, system or method listed is relevant to your study. If you are not sure if a list item applies to your research, read the appropriate section before selecting a response.

Materials & experimental systems

n/a	Involved in the study
☐	☒ Antibodies
☐	☒ Eukaryotic cell lines
☒	☐ Palaeontology
☒	☐ Animals and other organisms
☒	☐ Human research participants
☒	☐ Clinical data

Methods

n/a	Involved in the study
☒	☐ ChIP-seq
☐	☒ Flow cytometry
☒	☐ MRI-based neuroimaging

Antibodies

Antibodies used

All of the detailed information regarding the antibodies used in this study has been described in "Method" section. Here is the summary of the antibodies:
Flowcytometry: CD75 antibody (Abcam, ab77676, 1 µg for 1x106 cells), SUSD2-PE conjugated antibody (BioLegend, 327406, 5 µl for 1x106 cells), CD90-PE conjugated antibody (BD Pharmingen™, 561970, 1 µg for 1x106 cells), CD24-FITC conjugated antibody (BioLegend, 311104, 5 µl for 1x106 cells), CD317-APC conjugated antibody (BioLegend, 348410, 5 µl for 1x106 cells), and Alexa Fluor® 555 (Thermo Fisher Scientific, A-21426, 1:1000).

Whole-mount immunostaining: H3K27me3 antibody (Millipore, 07-449, 1:4000), CD75 antibody (Abcam, ab77676, 1 µg for 1x106 cells), CD90-PE conjugated antibody (BD Pharmingen™, 561970, 1 µg for 1x106 cells), TFE3 antibody (Sigma-Aldrich, HPA023881, 1:500), Oct-3/4 antibody (Santa Cruz Biotechnology, sc-5279, 1:200), KLF4 antibody (Santa Cruz Biotechnology, sc-20691, 1:300), Stella antibody (Millipore, MAB4388, 1:200), Alexa Fluor® 488 (Thermo Fisher Scientific, A21206, 1:1000), Alexa Fluor® 555 (Thermo Fisher Scientific, A-21426, 1:1000), and Alexa Fluor® 546 (Thermo Fisher Scientific, A10036, 1:1000).

In vitro differentiation: Cardiac Troponin T antibody (Thermo Fisher Scientific, MA5-12960, 5 µg/mL), HNF-4-alpha antibody (Abcam, ab92378, 1:100), and CYP3A4 antibody (Thermo Fisher Scientific, MA5-17064, 1:200), Pax-6 antibody (BioLegend, PRB-278P, 1:100), Alexa Fluor® 488 (Thermo Fisher Scientific, A21206, 1:1000), and Alexa Fluor® 546 (Thermo Fisher Scientific, A10036, 1:1000).

Validation

CD75 antibody (abcam, ab77676):
Mouse monoclonal [LN1] to CD75,
Host species: mouse
Applications: IHC-P, Flow Cyt, ICC/IF,
Species reactivity: human
Clonality: monoclonal
Clone number: LN1
Isotype: IgM

SUSD2-PE conjugated antibody (BioLegend, 327406):
Host species: mouse
Application: flowcytometry
Species reactivity: Human, African Green, Baboon, Cynomolgus
Clonality: monoclonal
Clone number: W5C5
Isotype: mouse IgG1, κ

CD90-PE conjugated antibody (BD Pharmingen™, 561970):
Host species: mouse
Applications: flow cytometry
Species reactivity: human, rhesus, cynomolgus, baboon, pig, dog
Clonality: monoclonal
Clone number: 5E10
Isotype: IgG1, κ

CD24-FITC conjugated antibody (BioLegend, 311104):
Host species: mouse
Applications: flowcytometry
Species reactivity: human, Cross-Reactivity: Chimpanzee
Clonality: monoclonal
Clone number: ML5
Isotype: mouse IgG2a, κ

CD317-APC conjugated antibody (BioLegend, 348410):
Host species: mouse
Applications: flowcytometry
Species reactivity: human, African Green, Baboon, Cynomolgus, Pigtail, Rhesus Macaque
Clonality: monoclonal
Clone number: RS38E

Isotype: mouse IgG1, κ

H3K27me3 antibody (Millipore, 07-449, 1:4000):
Host species: rabbit
Applications: ICC, IP, Mplex, WB, IHC
Species reactivity: human, mouse
Clonality: polyclonal
Isotype: IgG

TFE3 antibody (Sigma-Aldrich, HPA023881):
Host species: rabbit
Applications: immunofluorescence, immunohistochemistry
Species reactivity: rat, mouse, human
Clonality: polyclonal
Isotype: IgM

Oct-3/4 antibody (Santa Cruz Biotechnology, sc-5279):
Host species: mouse,
Applications: WB, IP, IF and ELISA,
Species reactivity: mouse, rat, human,
Clonality: monoclonal,
Clone number: C-10,
Isotype: IgG2b kappa

KLF4 antibody (Santa Cruz Biotechnology, sc-20691):
Host species: rabbit,
Applications: WB, IP, IF and ELISA,
Species reactivity: mouse, rat, human,
Clonality: polyclonal,
Clone number: H-180,
Isotype: IgG

Stella antibody (Millipore, MAB4388):
Host species: mouse,
Applications: ELISA, ICC,
Species reactivity: human, mouse,
Clonality: monoclonal,
Clone number: 3H5.2,
Isotype: IgG1

Cardiac Troponin T antibody (Thermo Fisher Scientific, MA5-12960):
Host species: mouse
Applications: ICC, IF, IHC, WB, Flow
Species reactivity: Avian, Dog, Chicken, Fish, Guinea pig, Human, Mouse, Pig, Rabbit, Rat
Clonality: monoclonal
Isotype: IgG1

HNF-4-alpha antibody (Abcam, ab92378):
Host species: rabbit
Applications: WB, IHC-P, Flow Cyt, ICC/IF
Species reactivity: human
Clonality: monoclonal
Isotype: IgG

CYP3A4 antibody (Thermo Fisher Scientific, MA5-17064):
Host species: mouse
Applications: Flow, ICC, IF, IHC, WB
Species reactivity: human
Clonality: monoclonal
Isotype: IgG1

Pax-6 antibody (BioLegend, PRB-278P):
Host species: rabbit
Applications: WB, IHC, IF
Species reactivity: Human, Mouse, Rat
Clonality: polyclonal
Isotype: IgG

Eukaryotic cell lines

Policy information about cell lines

Cell line source(s) | Human embryonic stem cell lines, H1 & H9, Wicell Research Institute Inc.

Authentication	We are certified and authenticized by WiCell to use H1 & H9 human embryonic cell lines.
Mycoplasma contamination	Cells were tested negative for mycoplasma contamination.
Commonly misidentified lines (See ICLAC register)	*Name any commonly misidentified cell lines used in the study and provide a rationale for their use.*

Flow Cytometry

Plots

Confirm that:

☒ The axis labels state the marker and fluorochrome used (e.g. CD4-FITC).

☒ The axis scales are clearly visible. Include numbers along axes only for bottom left plot of group (a 'group' is an analysis of identical markers).

☒ All plots are contour plots with outliers or pseudocolor plots.

☒ A numerical value for number of cells or percentage (with statistics) is provided.

Methodology

Sample preparation	All of the process and sample preparation for flow cytometry analysis are described in detail in Method section. Naïve and primed hPSCs from both static and stirred suspension cultures were dissociated into single cells using StemPro® Accutase® Cell Dissociation Reagent (Thermo Fisher Scientific, A1110501). To track the dynamics of SUSD2 and CD24 cell surface markers during primed to naïve conversion, the aggregates were collected from bioreactors every day from day 2 to day 6 of culture. For all other cell surface marker expression assessment, the aggregates of day four post-inoculation were collected. The aggregates were treated with Accutase for 10 min and pipetted to dissociate aggregates into single cells. The cells were washed with cell-specific medium (naïve or primed PSC medium) and were centrifuged at 200 g for 5 min. The cells were resuspended in 4% paraformaldehyde and were incubated for 15 min at RT. The fixed cells were then washed three times in 4 mL of PBS- (Thermo Fisher Scientific, 14190250) and were blocked in 10% BSA solution (BlockerTM BSA (10X) in PBS-, Thermo Fisher Scientific, 37525) at 37°C for 30 min. The primary antibodies were diluted in blocking solution and were added to the cells and incubated for 60 min at 4°C. The primary antibodies used for flow cytometry were CD75 antibody (Abcam, ab77676, 1 µg for 1x106 cells), SUSD2-PE conjugated antibody (BioLegend, 327406, 5 µl for 1x106 cells), CD90-PE conjugated antibody (BD Pharmingen™, 561970, 1 µg for 1x106 cells), CD24-FITC conjugated antibody (BioLegend, 311104, 5 µl for 1x106 cells), and CD317-APC conjugated antibody (BioLegend, 348410, 5 µl for 1x106 cells). After washing the cells with flow buffer containing 0.5% BlockerTM BSA (10X) in PBS-, the cells were incubated with secondary antibody Alexa Fluor® 555 (Thermo Fisher Scientific, A-21426, 1:1000) diluted in blocking solution for 60 min at 4°C. All antibodies were conjugated with fluorophores except CD75, therefore, the incubation time for the secondary antibody was only done for the CD75 antibody. The cells were then washed and resuspended in 200 µl flow buffer within a FACS tube and were analyzed using a BD FACSVantage SE System at the University of Calgary Flow Cytometry Facility. Gates were drawn based on isotype control. The gating strategy for each marker expression is shown in Supplementary Figures 5 and 7. The histogram of flow cytometry analysis was generated by FlowJo V10.1.3 software. 10.1.3.
Instrument	Flow cytometry data collection was done a BD FACSVantage SE System at the University of Calgary Flow Cytometry Facility.
Software	FlowJo V10.1.3 software. 10.1.3. was used to generate the histogram of flow cytometry analysis.
Cell population abundance	*Describe the abundance of the relevant cell populations within post-sort fractions, providing details on the purity of the samples and how it was determined.*
Gating strategy	Gating strategy is summarized in Supplementary Figures 5 and 7. The first gate (P1) identifies the cell population based on the cell size (FSC) and complexity of the cells (SSC). The next two gates (P2 & P3) enable the discrimination of cells versus debris and exclude the doublets and aggregates. The last gate demonstrates the expression level of the marker (CD75 or CD90) on single cells in the gated population. Gates were drawn based on isotype control.

☒ Tick this box to confirm that a figure exemplifying the gating strategy is provided in the Supplementary Information.
